# Antioxidants Attenuate Oxidative Stress-Induced Hidden Blood Loss in Rats

**DOI:** 10.4274/tjh.2016.0469

**Published:** 2017-12-01

**Authors:** Hong Qian, Tao Yuan, Jian Tong, Wen-shuang Sun, Jiajia Jin, Wen-xiang Chen, Jia Meng, Nirong Bao, Jianning Zhao

**Affiliations:** 1 Southeast University Nanjing General Hospital of Nanjing Military Command, Clinic of Orthopedics, Nanjing, China; 2 Nanjing University Faculty of Medicine, Jinling Hospital, Clinic of Orthopedics, Nanjing, China; 3 Nanjing University Faculty of Medicine, Nanjing General Hospital of Nanjing Military Command, Clinic of Orthopedics, Nanjing, China; 4 Southeast University Nanjing General Hospital of Nanjing Military Command, Department of Respiratory Medicine, Nanjing, China; 5 Southern Medical University Faculty of Medicine, Department of Orthopedics, Nanjing, China

**Keywords:** Hidden blood loss, Antioxidants, Proanthocyanidin, Hydrogen water

## Abstract

**Objective:**

Hidden blood loss (HBL), commonly seen after total knee or hip arthroplasty, causes postoperative anemia even after reinfusion or blood transfusion based on the visible blood loss volume. Recent studies demonstrated that oxidative stress might be involved in HBL. However, whether the antioxidants proanthocyanidin (PA) or hydrogen water (HW) can ameliorate HBL remains poorly understood. The aim of this study was to evaluate the effects of PA and HW on HBL.

**Materials and Methods:**

A rat HBL model was established through administration of linoleic acid with or without treatment with PA or HW. The levels of hemoglobin (Hb), red blood cell (RBC) count, superoxide dismutase (SOD) activity, glutathione peroxidase (GSH-PX) activity, malondialdehyde (MDA), and ferryl Hb were measured.

**Results:**

RBC and Hb values as well as the activity of SOD and GSHPX were reduced after administration of linoleic acid, which was ameliorated by treatment with PA or HW. In addition, the quantity of MDA was significantly decreased with the administration of PA or HW.

**Conclusion:**

PA and HW could ameliorate HBL in a rat model by reducing oxidative stress, suggesting that they might be used as a novel therapeutic approach in the prophylaxis or treatment of HBL in clinics.

## Abstract (tr)

**Amaç:**

Total diz veya kalça artroplastisinden sonra yaygın olarak görülen gizli kan kaybı (GKK), görülebilir kan hacmi kaybına dayanan reinfüzyon veya kan nakli sonrasında bile postoperatif anemiye neden olur. Son yıllarda yapılan çalışmalar oksidatif stresin GKK’yla ilişkili olabileceğini göstermiştir. Bununla birlikte, proantosiyanidin (PA) veya hidrojenli su (HS) antioksidanlarının GKK’yi iyileştirip iyileştirmediği anlaşılamamıştır. Bu çalışmanın amacı PA ve HS’nin GKK üzerindeki etkilerini değerlendirmektir.

**Gereç ve Yöntemler:**

PA veya HS ile muamele edilmiş veya edilmemiş olan linoleik asit uygulaması ile bir sıçan GKK modeli oluşturulmuştur. Hemoglobin (Hb), kırmızı kan hücresi sayısı (RBC), süperoksit dismutaz (SOD) aktivitesi, glutatyon peroksidaz (GSH-PX) aktivitesi, malondialdehit (MDA) ve ferril Hb düzeyleri ölçüldü.

**Bulgular:**

PA veya HS verilen linoleik asit uygulaması ile RBC ve Hb değerlerinin yanı sıra SOD ve GSH-PX aktiviteleri azaltıldı. Buna ek olarak, PA veya HS uygulaması ile MDA miktarı önemli ölçüde azaldı.

**Sonuç:**

PA ve HS bir sıçan modelinde oksidatif stresi azaltarak GKK’yi düzeltebildiğinden, kliniklerdeki GKK profilaksisi veya tedavisinde yeni bir terapötik yaklaşım olarak kullanılabileceklerini düşündürmektedir.

## INTRODUCTION

Artificial joint replacements are widely employed to alleviate pain and improve the quality of patients’ lives [1]. The rates of primary and total hip arthroplasty (THA) and total knee arthroplasty (TKA) are estimated to increase by 174%-673% by 2030 as the population ages [2]. However, hidden blood loss (HBL) predominantly occurs after artificial joint replacement, such as in cases of TKA and THA [4]. The consequential acute anemia and transfusions are major concerns for joint surgeons.

The pathogenesis of HBL is very complicated, involving several factors. A recent study demonstrated that free fatty acids (FFAs) generated from fatty emboli in the blood circulation are responsible for HBL through peroxidation injury of membrane molecules of red blood cells (RBCs) and hemoglobin (Hb) [4]. In addition, antioxidants administered intra- or postoperatively are predicted to play a protective role in erythrocyte oxidation and potentially reduce the volume of HBL after arthroplasty, suggesting that oxidation might be involved in the pathogenesis of HBL. Consistent with this, our previous study also demonstrated that FFAs can induce RBC and Hb damage via reactive oxygen species (ROS) toxicity in vivo [5]. As a natural antioxidant extract from grape seeds, proanthocyanidin (PA) possesses a wide range of bioavailability [6]. PA exhibits higher protective effects against DNA damage and lipid peroxidation induced by ROS compared with β-carotene, vitamin C, and vitamin E [7]. PA is a safe and effective bioavailable antioxidant and ROS scavenger, which is used for the treatment of ischemia/reperfusion injuries of multiple organs, malignant tumor progression, carcinogenesis, gastrointestinal disorders, and Parkinson and Alzheimer disease [6].

As a new antioxidant, hydrogen water (HW) has also been applied to prevent and treat oxidative stress-associated illnesses using the establishment of animal models [8,9,10]. HW has been proven to selectively remove strong oxidants including peroxynitrite and hydroxyl radicals. Alternatively, ROS play a physiological role in preventing cells from experiencing oxidative stress [11].

Considering the role of oxidative stress in the pathogenesis of HBL, whether PA and/or HW as antioxidants ameliorate HBL remains poorly understood. The objective of this study was to evaluate the effect of PA and HW on HBL as well as to compare their protective effects by measuring the levels of Hb, RBC count, superoxide dismutase (SOD), glutathione peroxidase (GSH-PX), malondialdehyde (MDA), and ferryl Hb.

## MATERIALS AND METHODS

### Animals

Forty 10-week-old male Sprague-Dawley rats weighing 250±20 g were obtained from the Nanjing University Model Animal Research Center. All animals were fed daily with rat feed and potable water or HW under appropriate laboratory conditions at 24 °C with a 12-h light/dark cycle. The animals were randomly assigned into four groups (n=10 per group). Experimental procedures were performed strictly according to the Guide for the Care and Use of Laboratory Animals proposed by the National Research Council in 1996. All animals were properly monitored. Animal ethics approval was obtained for this research. All experimental procedures conducted complied with the guidelines of the National Institutes of Health Guide for the Care and Use of Laboratory Animals and the Institutional Care and Use Committee of Nanjing University. Preoperatively, all animals were anesthetized via ether inhalation.

### Instruments and Reagents

Instruments used included a hematology analyzer (SYSMEX XE-5000, Kobe, Japan), centrifuge (Hermle Universal Centrifuge Z323, Gosheim, Germany), microplate reader (Bio-Rad 680, Hercules, CA, USA), polarizing microscope (Nikon Eclipse 50I, Tokyo, Japan), spectrophotometer (Hewlett Packard 8453 UV-visible diode array spectrophotometer, Palo Alto, CA, USA), HW-generating apparatus (Bio Coke Laboratory, Tokyo, Japan), and hydrogen sensor (DHS-001, ABLE, Tokyo, Japan).

The concentration of MDA and the activities of SOD and GSH-PX were measured with commercially available assay kits (Nanjing Jiancheng Bioengineering Institute, Nanjing, China). Linoleic acid was purchased from Sigma-Aldrich (St. Louis, MO, USA). PA was purchased from Shanghai Aladdin Bio-Chem Technology Institute (Shanghai, China). HW was prepared by dissolving H2 gas in drinking water under high pressure of 0.4 MPa using the HW-generating apparatus. Rats were supplied with HW (0.7 mM) through a closed glass vessel (300 mL) equipped with an outlet line containing 2 ball bearings to prevent water degassing. The H2 concentration of HW was detected with a hydrogen sensor (Unisense, Aarhus, Denmark).

### Experimental Protocol and Drugs

The procedures below were performed on the rats in all four groups and the dose used was selected as previously described. The control group (CON) rats were given potable water and injected with ethanol alone (0.5 mL, 20%) via intravenous administration into the tail vein after 2 weeks of feeding.

The linoleic acid (LIN) group animals (receiving LIN) received potable water and were injected with 0.5 mL of 60 mmol/L linoleic acid diluted in 20% ethanol by intravenous administration into the tail vein after 2 weeks of feeding.

The LIN+PA group received a 100 mg/kg dose of PA diluted with potable water daily [12,13] and was injected with 0.5 mL of 60 mmol/L linoleic acid diluted in 20% ethanol by intravenous administration into the tail vein after 2 weeks of feeding [5].

The LIN+HW group received HW daily and was injected with 0.5 mL of 60 mmol/L linoleic acid diluted in 20% ethanol by intravenous administration into the tail vein following 2 weeks of feeding [5].

During all treatments, rats were monitored daily and were weighed one to six times per day until the end of the experiment. None of the rats had any notable discomfort throughout the experiment.

### Routine and Biochemical Analysis of Blood

Blood samples were taken from the caudal vein under anesthesia (0.5 mL each time) at the beginning of the injection and 24, 48, and 72 h following administration. RBC, hematocrit, and Hb levels were detected with a hematology analyzer immediately after sampling collection. Morphological changes of blood cells were observed following Wright’s staining under a polarizing microscope.

The remaining blood samples were centrifuged and stored at 80 °C for subsequent biochemical analysis. MDA, T-SOD, and GSH-PX activities were measured by spectrophotometer. The absorbance values were detected at 532 nm, 550 nm, and 412 nm wavelengths [12]. Spectral changes of Hb in the LIN and LIN+PA groups were quantitatively measured by spectrophotometer. Hb at a concentration of 10 mM was mixed with 0.1 M sodium phosphate buffer containing 100 mM DTPA. All experimental procedures were conducted at 25 °C [14].

### Statistical Analysis

Statistical analysis was performed using SPSS 19.0. All data were expressed as mean ± standard deviation. The Kolmogorov-Smirnov test was performed and we concluded that the observed data were from a population specified by normal distribution. One-way analysis of variance (ANOVA) was performed followed by the Tukey test. p<0.05 was considered statistically significant.

## RESULTS

Daily consumption of water and body weight among all groups were monitored. Rats in the CON group consumed 20.0±3.5 mL of potable water daily, while the LIN group consumed 21.0±2.7 mL of potable water daily. In the LIN+PA group, daily consumption of PA solution was 22.0±2.4 mL, while the LIN+HW group consumed 24.0±3.4 mL of HW daily. Water consumption and body weight did not significantly differ among the four groups.

### Routine Blood Tests

**Figure 1:**
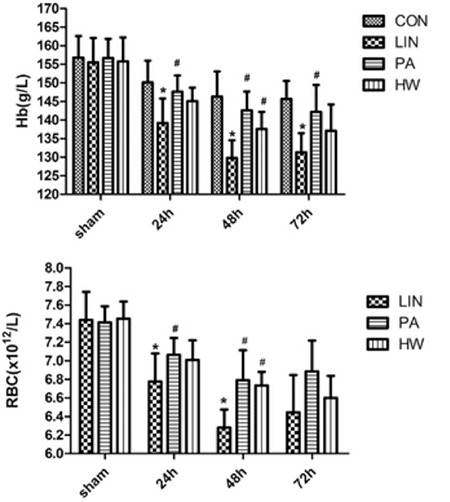
Changes of hemoglobin and red blood cell levels with time between control (sham) group and experimental groups. Values are presented as the mean ± standard deviation, n=10 for all groups. *Compared with the control group, p<0.05, #Compared with the linoleic acid group, p<0.05. LIN: Linoleic acid, PA: proanthocyanidin, HW: hydrogen water, RBC: red blood cell, Hb: hemoglobin.

Before linoleic acid administration, no significant differences were observed in RBC and Hb levels among the four groups. After administration of a dose of 0.5 mL of 60 mmol/L linoleic acid, RBC and Hb levels significantly changed compared with the control group (Figure 1), which showed that an in vivo HBL model had been established successfully. We further analyzed the RBC and Hb levels of the LIN+PA and LIN+HW groups compared to those of the LIN group. After 24 h of administration, the Hb and RBC levels had decreased to different extents in the three experimental groups. In the LIN group, the RBC and Hb values were reduced by (0.66±0.34)×1012/L and 16.3±8.25 g/L, and in the LIN+PA group those values were decreased by (0.35±0.1)×1012/L and 9.1±4.01 g/L, respectively. A significant difference was noted in the changes between the LIN and LIN+PA groups. After 48 h of administration, the changes of RBC and Hb levels of the LIN group and the LIN+PA group were still significantly different. In the LIN+HW group, we found the RBC and Hb values decreased by (0.45±0.22)×1012/L and 10.7±3.56 g/L after 24 h, respectively, with a tendency of alleviation of the reduction of RBC and Hb levels. After 48 h, the decreases of RBC and Hb (respectively (0.72±0.23)×1012/L and 18.2±5.85 g/L) in the LIN+HW group were significantly different compared to those of the LIN group (respectively (1.15±0.48)×1012/L and 25.7±8.38 g/L).

### Oxidative Stress Markers

**Figure 2:**
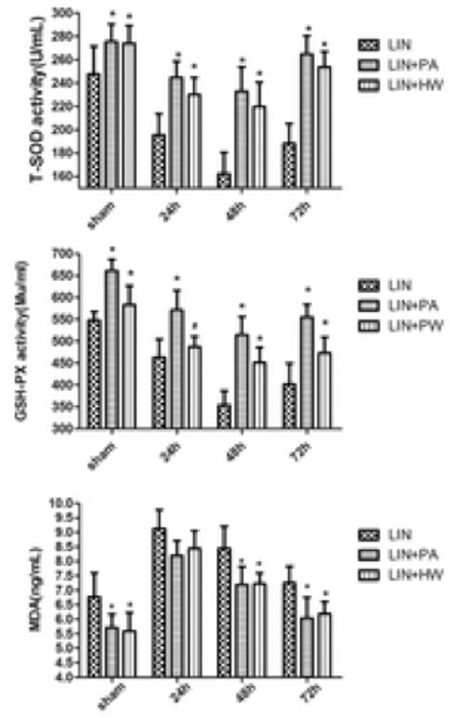
Changes in T-superoxide dismutase, glutathione peroxidase, and malondialdehyde among the linoleic acid, linoleic acid+proanthocyanidin, and linoleic acid+hydrogen water groups. Values are presented as the mean ± standard deviation, n=10 for all groups. *Compared with the control group, p<0.05. LIN: Linoleic acid, PA: proanthocyanidin, HW: hydrogen water, SOD: superoxide dismutase, GSH-PX: glutathione peroxidase, MDA: malondialdehyde.

The activities of SOD and GSH-PX in the LIN group significantly declined after 24 h of administration, reached the lowest levels after 48 h, and had mild increases after 72 h. Both the LIN+PA and the LIN+HW group showed a similar variation tendency in these two markers. However, the SOD and GSH-PX activities in groups LIN+PA and LIN+HW were both obviously higher than those of the LIN group at each time point (Figure 2).

The MDA concentration in the LIN group reached a peak after 24 h and then started to decrease slowly. The LIN+PA and LIN+HW groups also displayed a similar changing pattern in MDA level. However, both SOD and GSH-PX activities in groups LIN+PA and LIN+HW were consistently lower than those of the LIN group (Figure 2).

**Figure 3:**
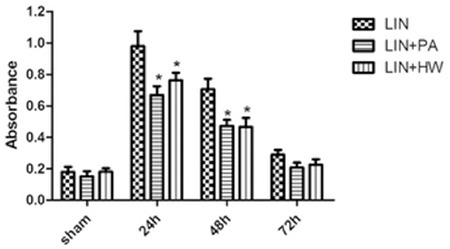
Changes in absorbance at 425 nm among the linoleic acid, linoleic acid+proanthocyanidin, and linoleic acid+hydrogen water groups. Values are presented as the mean ± standard deviation, n=10 for all groups. *Compared with the control group, p<0.05. LIN: Linoleic acid, PA: proanthocyanidin, HW: hydrogen water.

Ferryl Hb was present and formed by reacting with H2O2, which was confirmed by the characteristic absorbance band around 620 nm via the reaction with sulfide ions [15]. The effect of linoleic acid upon the hemolysis of RBCs, either by itself or in conjunction with ROS, can be utilized to assess the severity of oxidative injury of erythrocytes [16]. Blood samples were collected from these three groups before administration and every 24 h thereafter. Absorbance peak values were detected at a wavelength of approximately 425 nm, consistent with the Soret peak of ferryl Hb (Figure 3).

### Histologic Investigations

**Figure 4:**
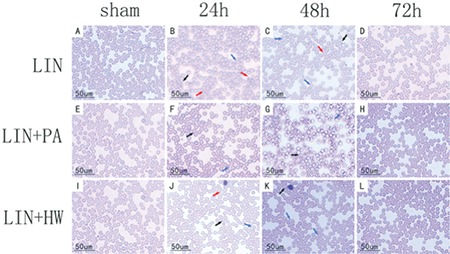
Protective effects of proanthocyanidin and hydrogen water on red blood cells. Blood samples were collected before administration and then every 24 h thereafter. Stains were added to the blood smears to observe erythrocyte morphological changes. Images are magnified at 400x. After 24 h, cell morphology was obviously changed in the linoleic acid group (A-D) with a large number of red blood cells shrunken (black arrows), deformed (blue arrows), and even ruptured (red arrows); in contrast, in the linoleic acid+proanthocyanidin group (E-H) and LIN+HW group (I-L), ruptured cells could hardly be identified. LIN: Linoleic acid, PA: proanthocyanidin, HW: hydrogen water.

In the LIN group, a number of shrunken, deformed, and ruptured blood cells were seen compared with the control group and the morphological changes were the most distinct after 24 h of administration. In contrast, we could also identify some shrunken and deformed RBC in groups LIN+PA and LIN+HW, but ruptured blood cells could hardly be found in those two groups (Figure 4).

## DISCUSSION

Many recent studies have investigated the pathophysiological mechanisms and therapeutic strategies for HBL [17,18,19], but to our knowledge, our team provided the first evidence that oxidative stress can lead to HBL [10] and that antioxidant treatment with PA or HW ameliorated HBL, suggesting they may represent a possible therapeutic choice for HBL in clinical practice.

HBL is a severe complication after TKA and THA [20]. Although several theories concerning the mechanisms of HBL have been proposed, no theory is convincing enough to explain the pathological mechanism. Pattison et al. [21] proposed that hemolysis may partly contribute to postoperative loss, but they did not provide a pathological mechanism. Faris et al. [22] demonstrated that hemolysis was detected after reinfusion with an average volume of 1.3 L of blood, but hemoglobinuria did not occur due to the activity of Hb. In contrast, Shen et al. [23] showed that no statistical significance was observed in HBL between the reinfusion and non-reinfused groups. Li et al. [24] reported that administration of a tourniquet could significantly increase HBL in their study, but as much as 600 mL of HBL can be detected without using a tourniquet. Moreover, the theory of the “third compartment” was proposed to explain the mechanism underlying HBL. Erskine et al. [25] reported that unexplained blood loss was completely due to perioperative bleeding, probably into the tissue compartments. However, it is not reasonable that the bleeding would be “pressing” into tissue compartments because of commonly used techniques, such as negative pressure drainage and pressure dressing. Therefore, subsequent investigation is required to fully unravel the mechanisms underlying HBL.

The increased intramedullary pressure in TKA and THA plays a vital role in the pathogenesis of fatty metabolism [26,27]. In addition to the clinical association between fatty emboli and cardiopulmonary function, the metabolites of fatty emboli, FFAs, can stimulate ROS production in neutrophils [28] and exert a negative biological effect on erythrocytes. After ROS were stimulated and the oxidants accumulated, osmotic fragility of RBCs increased through oxidizing polyunsaturated fatty acids derived from the RBC membranes [29] and cytosolic ferrous Hb [30].

Given the critical role of ROS in the damage or injury of RBCs, this study investigated the antioxidant effect of PA and HW on linoleic acid-induced oxidative stress by measuring GSH-Px, SOD, and MDA. Our results showed that SOD and GSH-Px activities were increased in the experimental groups with the use of PA or HW, and the SOD and GSH-Px activities of each experimental group were significantly decreased after 24 h of LIN administration, indicating that linoleic acid plays a vital role in promoting oxidation responses in the body and reducing SOD and GSH-Px activity. In the LIN+PA and LIN+HW groups, SOD and GSH-Px showed significantly elevated activities compared with the LIN group. These findings can be interpreted as PA and HW exhibiting a positive effect on SOD and GSH-Px activity. The amount of MDA was significantly decreased due to the effect of linoleic acid, suggesting the presence of oxidative stress in the culture medium. In this study, the quantity of MDA was significantly decreased with the administration of PA or HW [9,12], consistent with previous studies showing that the elevation of MDA level induced by lipid peroxidation was counteracted by the administration of PA or HW. Although the present study indicates that PA possesses higher anti-HBL effects compared with HW, no significant variation occurred in our study considering the dosage and duration of PA administration.

Oxidative injury changes the structure and function of Hb, leading to Hb denaturation and precipitation. The resultant product is known as methemoglobin [19]. Hydrophilic hydrogen peroxide is capable of directly penetrating the RBC membrane and oxidizing Hb into ferryl Hb [20]. The heme proteins oxidized into the ferryl species by peroxides are widely regarded as the initiators of a variety of lipid peroxidation and lipid pseudo-peroxidase responses [21]. Hypochlorous acid can oxidize glutathione and membrane protein-SH groups and elevates the osmotic fragility. In addition, it also induces cell membrane deformation via lipid oxidation [3]. Multiple investigations have indicated that ferrous Hb can be oxidized into ferryl Hb by H2O2 and hypochlorous acid. Ferryl Hb loses the capacity of carrying oxygen. Nevertheless, GSH-Px is able to decrease the formation of methemoglobin by 93% when Hb is oxidized by H2O2 [22], highlighting the pivotal role of linoleic acid in mediating Hb oxidation and subsequent cross-linking of the oxidation-reduction responses.

Several limitations have to be acknowledged in this study. First, our studies suggest that FFAs could cause HBL, which could be ameliorated through treatment with antioxidant drugs, but we cannot draw the conclusion that oxidative stress produced by FFAs leading to the toxicity of RBCs is the only pathophysiological mechanism underlying postoperative blood loss. Second, the appropriate therapeutic dose and timing of PA and HW administration and the combination therapy of these two drugs need further investigation. The significance of the current experiment is that HBL induced by ROS increase can be counteracted by antioxidant therapy.

## CONCLUSION

In conclusion, PA and HW could ameliorate HBL in a rat model by reducing oxidative stress, suggesting they might be used as novel therapeutic approaches in the prophylaxis or treatment of HBL in clinical practice.
